# Integrated Multiparametric MRI Scoring System-Based Approach in Differentiating Adrenal Adenoma From Other Adrenal Lesions

**DOI:** 10.7759/cureus.84564

**Published:** 2025-05-21

**Authors:** Fatma M Al Hajri, Ishaq Al Salmi

**Affiliations:** 1 Nuclear Medicine, The Royal Hospital, Muscat, OMN; 2 Radiology, The Royal Hospital, Muscat, OMN

**Keywords:** adrenal adenoma, adrenal incidentalomas, adrenal lesions, mri adrenal, scoring system

## Abstract

Introduction

Advancements in imaging technology have led to increased detection of adrenal lesions.Differentiating adrenal adenomas from other lesions is crucial for therapy guidance and avoiding unnecessary interventions. The study aims to develop and assess the diagnostic efficacy of a holistic multiparametric magnetic resonance imaging (mpMRI) scoring system in distinguishing adrenal adenomas from other lesions.

Methods

After obtaining ethical approval, a retrospective observational study was conducted at a single center. It included 77 patients who underwent adrenal MRI with confirmed diagnoses via pathology or typical computed tomography (CT)/MRI findings. A fellowship-trained body imaging radiologist reviewed the MRI examinations, assessing key characteristics including size, T2-weighted imaging (T2WI) signal intensity (SI), chemical shift imaging (CSI), and contrast washout at five minutes. Sensitivity, specificity, positive predictive value (PPV), and negative predictive value (NPV) were calculated. Accordingly, the scoring system was created, and a total score of 3 or more was considered diagnostic for adrenal adenoma.

Results

Using a score of 3 or higher as a diagnostic threshold for adrenal adenoma, the multiparametric MRI (mpMRI) scoring system demonstrated high diagnostic accuracy, yielding a sensitivity of 98.6%, specificity of 93.8%, PPV of 98.6%, and NPV of 93.8%. Interestingly, homogeneous T2-weighted signal intensity emerged as a highly specific and distinguishing characteristic of adrenal adenomas, reinforcing its diagnostic value in differentiating them from other adrenal lesions.

Conclusion

Implementing the mpMRI scoring system to differentiate adrenal adenomas from other adrenal lesions has significantly increased the diagnostic yield of adrenal MRI compared to the old approach that solely depended on the presence of microscopic fat. However, a key limitation of this study is the reliance on imaging findings rather than histopathological confirmation for most cases. This reflects real-world clinical practice but highlights the need for further prospective validation.

## Introduction

Incidental detection of adrenal lesions

Advancements in imaging technology have led to an increased detection rate of adrenal lesions. Notably, adrenal lesions discovered incidentally during cancer workup were found in approximately 5%-8% of patients who underwent computed tomography (CT). Furthermore, among patients with a known primary malignancy who underwent CT, adrenal lesions were identified in approximately 9%-13% of cases, with metastatic lesions accounting for approximately 26%-36% of these cases [1,2]. Consequently, accurately identifying adrenal adenomas from other adrenal lesions is crucial to ensure proper treatment decisions and avoid unnecessary biopsies or repeated follow-up tests [1,2].

Limitations of CT imaging

Differentiating adrenal adenomas from other adrenal lesions relies on computed tomography (CT) and magnetic resonance imaging (MRI) as the preferred imaging modalities. However, there are certain limitations associated with these techniques that warrant improvement. Specifically, non-enhanced CT images reveal that only approximately two-thirds of adrenal adenomas exhibit a density of less than 10 Hounsfield units (HU). At the same time, the remaining one-third are lipid-poor adenomas with a density exceeding 10 HU. Furthermore, the washout range observed in adrenal adenomas overlaps with that observed in hypervascular metastases and approximately one-third of pheochromocytomas, further complicating the differentiation process [3].

Chemical shift imaging (CSI) pitfalls

Chemical shift imaging (CSI) is the primary magnetic resonance (MR) sequence for assessing adrenal lesions. It functions by detecting the presence of microscopic fat within adrenal adenomas. The sensitivity of CSI in identifying adrenal adenomas ranges from 81% to 100%, while its specificity ranges from 94% to 100%, as reported in the literature [4,5]. A significant limitation of chemical shift imaging (CSI) is its inability to detect approximately 15%-20% of adrenal adenomas classified as lipid-poor adenomas. Additionally, positive chemical shift artifacts can be observed not only in adrenal adenomas but also in other lesions, including adrenocortical carcinoma (ACC), pheochromocytoma, clear cell renal cell carcinoma (ccRCC), and metastases of hepatocellular carcinoma (HCC) containing fat [6], which further complicates the differentiation between these various lesions based solely on CSI findings.

Role of dynamic contrast-enhanced MRI

Previous literature has highlighted specific characteristics of early dynamic gadolinium-enhanced adrenal MRI, including the measurement of time to peak (TTP). Malignancies tend to exhibit a slower time to enhance compared to adenomas, indicating a delay, in contrast to uptake in malignancies compared to benign adenomas [7]. This difference in TTP can serve as a distinguishing feature between adrenal malignancies and adenomas during the early dynamic phase of gadolinium-enhanced MRI.

Need for an integrated mpMRI scoring system

Given the overlapping features and limitations of individual MRI parameters, there is a growing need for a structured, multiparametric approach to improve diagnostic accuracy. Therefore, this retrospective, single-center study aims to develop and evaluate the effectiveness of an integrated multiparametric MRI (mpMRI) scoring system in distinguishing adrenal adenomas from other adrenal lesions.

## Materials and methods

Subjects

All gadolinium-enhanced adrenal MRI scans performed during January 2010 and December 2020 were retrieved from the picture archiving and communication system (PACS) and evaluated by a board-certified radiologist.

The study included all patients who underwent an adrenal MRI between 2010 and 2020 and had a confirmed diagnosis of an adrenal lesion. A confirmed diagnosis (gold standard) was defined as follows: histopathology, presence of microscopic fat on computed tomography (CT) or chemical shift imaging (CSI), typical washout pattern on CT without a fat-containing primary tumor elsewhere in the body, two years of stability in size and appearance, or a combination of the above criteria.

**Figure 1:**
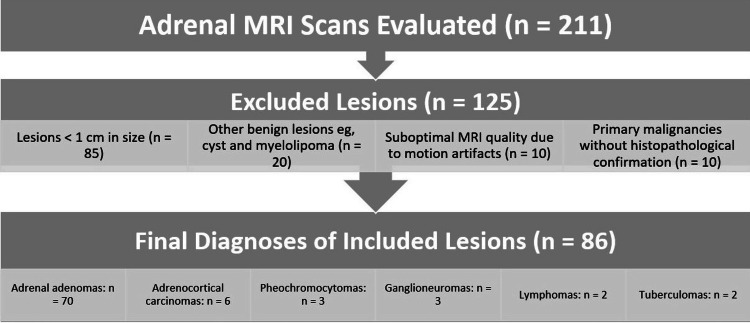
Flowchart summarizing the inclusion and exclusion criteria for all adrenal lesions evaluated by adrenal MRI from 2010 to 2020 MRI: magnetic resonance imaging

A total of 125 adrenal lesions were excluded from the research due to specific criteria. Eighty-five lesions measuring less than 1 cm were omitted because their small size hindered the comprehensive assessment of essential imaging attributes and precise delineation of regions of interest (ROIs). An additional set of 20 lesions was excluded, as their benign character was established solely by assessing T2-weighted imaging (T2WI), encompassing conditions such as cysts, pseudocysts, and myelolipomas. Ten cases, in which patients were already diagnosed with primary malignancies such as hepatocellular carcinoma and renal cell carcinoma, were not considered, as there was no histopathological confirmation for their adrenal lesions. Furthermore, 10 lesions were excluded from the study due to suboptimal MRI quality resulting from motion artifacts (Figure 1).

MRI protocol

A 1.5 T Signa Architect MRI scanner (GE Healthcare, Waukesha, WI) was used for the adrenal MRI examinations. The scans were performed using a phased-array torso coil. The following MRI protocol was employed: axial T1-weighted in and out of phase turbo-field echo (TFE), axial T2-weighted spectral attenuated inversion recovery (SPAIR) sequence, axial T2-weighted long-time echo (TE), coronal T2-weighted turbo spin echo (TSE), axial diffusion-weighted images, and axial T1-weighted 3D gradient-echo sequence with fat suppression before and after the administration of contrast. All images were acquired with a slice thickness of 4 mm, an interslice gap of 0.4 mm, and a matrix size of 256 × 256. Image quality was assessed and standardized based on signal-to-noise ratio (SNR) and absence of motion artifacts.

A real-time bolus-tracking method was utilized to capture the arterial phase. Imaging was initiated with a delay of 8-10 seconds from the trigger point when the contrast material reached the celiac trunk. Breath-hold instructions were given to the patient, and the acquisition of 3D gradient-echo images was completed within a single breath-hold, which took approximately 17-18 seconds. Venous phase images were acquired 45-60 seconds after contrast administration, followed by continuous dynamic imaging up to 120 seconds. Additionally, delayed phase images were obtained at five minutes post-contrast administration. Gadolinium-based contrast agent gadoterate meglumine (DOTAREM) was injected using a power injector at 2 mL/s with a 0.05 mmol/Kg dosage, followed by a 20 mL normal saline bolus.

Image analysis and scoring system

A single fellowship-trained body imaging radiologist with five years' experience, blinded to the clinical information, reviewed the MR images of the adrenal lesions. Each lesion was scored based on its imaging features.

The multiparametric MRI (mpMRI) scoring system was empirically derived based on a comprehensive review of the literature and the diagnostic characteristics observed in this study's dataset. It was then retrospectively applied to evaluate its performance in differentiating adrenal adenomas from other adrenal lesions. The size of the lesions was determined by measuring the most significant dimension observed on axial T1-weighted pre-contrast images or coronal T2-weighted images.

**Figure 2:**
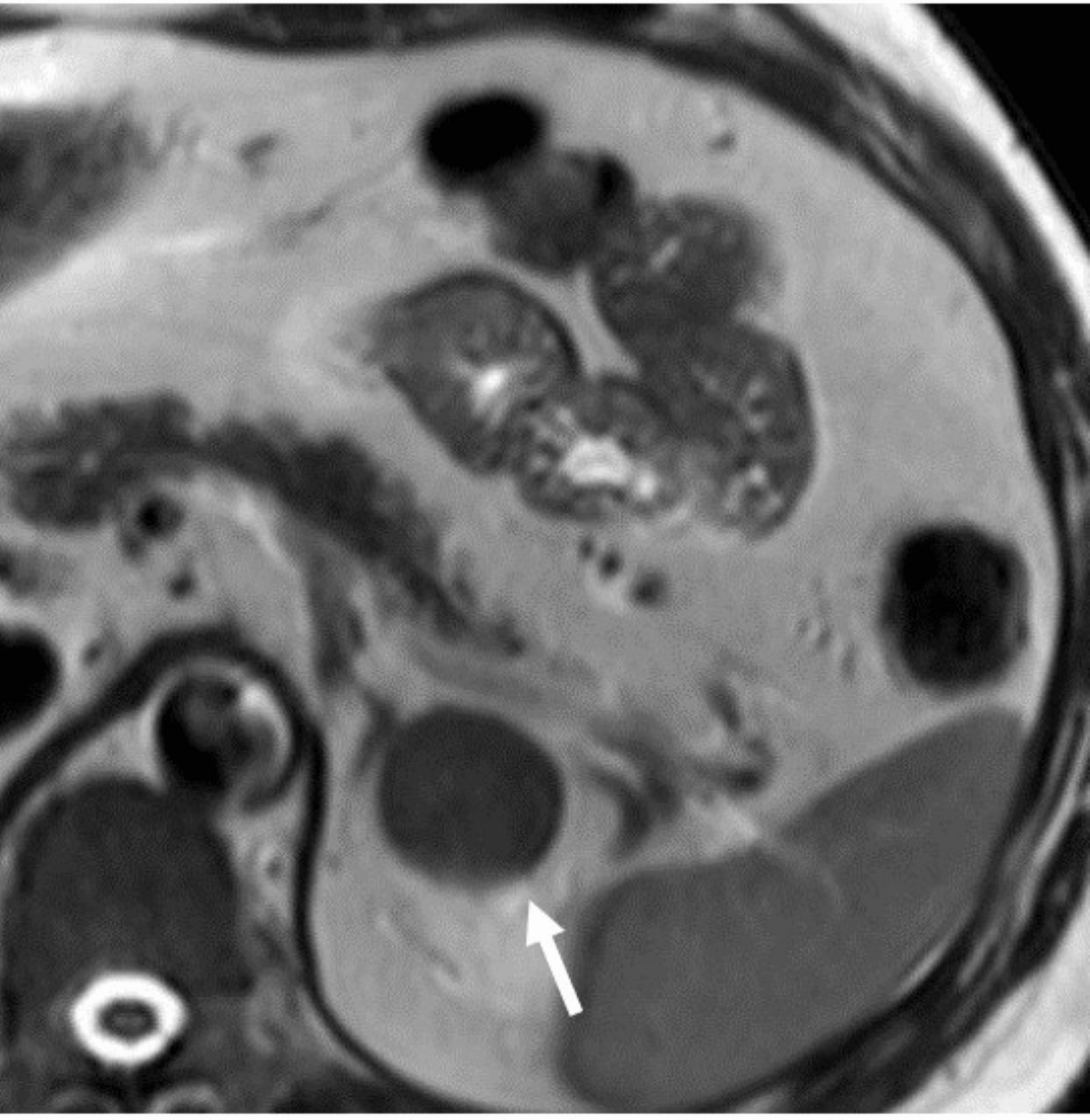
Axial long TE, T2-weighted MRI for a 33-year-old man with incidental adrenal adenoma showing homogeneous signal intensity (white arrow) TE: time echo, MRI: magnetic resonance imaging

The qualitative evaluation of T2-weighted images primarily focused on the overall signal intensity (SI) to determine whether the lesion exhibited homogeneous or heterogeneous characteristics. Lesions displaying a homogeneous T2 signal intensity were assigned a score of 1 (Figure 2), while those with heterogeneous features received a score of 0.

Qualitative assessment of post-contrast images involved evaluating enhancement timing (early or late) and the pattern of enhancement, which was categorized into five main patterns: homogeneous, punctate, peripheral, heterogeneous enhancement, or no enhancement [8].

Quantitative assessment was performed by selecting a region of interest (ROI) that was uniform in size across all pre- and post-contrast dynamic images. Several parameters were measured within the ROI, including plain signal intensity (SI), time to peak enhancement, post-contrast signal intensity at one, three, and five minutes after contrast administration, maximum SI in post-contrast images, washout SI at five minutes (compared to the SI value at one minute), and maximum SI washout (compared to the maximum SI value at any time between one and five minutes). Specific considerations were taken into account when choosing the ROIs, such as covering two-thirds of the lesion, avoiding edges to minimize partial volume averaging, and measuring the area with the highest enhancement if the lesion exhibited heterogeneity [8].

The scoring system was developed based on a comprehensive review of the literature on valuable adrenal MRI characteristics. These characteristics include lesion size, signal drop-out on out-of-phase (OOP) images, time to peak enhancement, maximum washout signal intensity (SI), and washout SI at five minutes.

A positive score is assigned to the presence of an imaging feature associated with an adrenal adenoma. If there is a strong association, it is given a score of +2, while a mild to moderate association is assigned a score of +1. If the absence of an imaging feature does not exclude or significantly reduce the likelihood of an adenoma, it is assigned a score of 0 (zero). Conversely, if there is a strong negative association between an imaging feature and the diagnosis of adrenal adenoma, the presence of that feature is given a score of -2 (minus two). The details of the scoring system can be found in Table 1.

**Table 1:** Scoring system employed in the study Each adrenal lesion was assigned scores based on specific criteria. For example, lesions with a size smaller than 5 cm received a score of 1, while lesions equal to or larger than 5 cm were assigned a score of -2. CSI: chemical shift imaging, SI: signal intensity, T2WI: T2-weighted imaging, WO: washout

Image characteristics	Score value	
Size	(<5 cm) 1	(≥5 cm) -2
CSI	(+ve) 2	(-ve) 0
SI on T2WI	(Homogeneous) 1	(Heterogeneous) 0
Time to peak	(<80 seconds) 1	(≥80 seconds) 0
5-minute WO SI	(>25%) 1	(≤25%) 0

The size of adrenal lesions is crucial in distinguishing between benign and malignant lesions. Various cutoff values have been reported in the literature, typically ranging from 4 to 6 cm. Lesions more significant than these cutoff values are generally considered more likely to be malignant, and surgical resection is often recommended [9-11]. For this study, a cutoff value of 5 cm was chosen to differentiate between benign and potentially malignant adrenal lesions.

This study assessed the signal intensity (SI) of chemical shift imaging (CSI) subjectively and objectively. The subjective evaluation involved visually examining the gross appearance of the lesion and determining whether there was a significant drop in SI between in-phase and out-of-phase images. An objective assessment was performed by drawing a circular region of interest (ROI) within the lesion and measuring the SI on both in-phase and out-of-phase images. The same precautions and considerations discussed during the ROI enhancement measurement were considered during the SI measurement.

The signal intensity index (SI index) was determined using the following formula: ((SI on in-phase imaging - SI on out-phase imaging) / (SI on in-phase imaging)) × 100% [6]. A positive SI index was defined as being greater than 16.5% [7].

In the published literature, there was no consensus on the cutoff values for time to peak (TTP), maximum washout SI, and five-minute washout SI. Therefore, the proposed cutoff values were selected based on the study sample to achieve the highest sensitivity and specificity.

In this study, a diagnostic threshold of 3 or more was established as indicative of an adrenal adenoma. Adrenal lesions with a total score equal to or higher than 3 were classified as adrenal adenomas based on the scoring system criteria utilized in the study.

Statistical analysis

Statistical analysis was conducted using IBM SPSS Statistics version 29 (IBM Corp., Armonk, NY). Descriptive data were presented as means with standard deviations for continuous variables and frequencies with percentages for categorical variables. Diagnostic performance measures, including sensitivity, specificity, positive predictive value (PPV), and negative predictive value (NPV), were calculated with corresponding 95% confidence intervals (CIs) to assess the effectiveness of individual imaging parameters and the overall multiparametric MRI (mpMRI) scoring system.

Ethical aspect

Ethical clearance for this study was obtained from the Scientific Research Committee at The Royal Hospital on February 16, 2021 (approval number: SRC#25I2021). The ethics committee waived written informed consent due to the study's retrospective nature and the use of anonymized patient data. This study complied with the principles outlined in the Declaration of Helsinki.

## Results

Demographics and lesion characteristics

The study included 86 adrenal lesions in 77 subjects. Of the 77 patients, 22 were men (29%), and 55 were women (71%), with ages ranging from 19 to 84 and a mean age of 39. Bilateral adrenal lesions were found in nine patients.

Out of the total of 86 adrenal lesions, only 18 had a confirmed diagnosis based on histopathology, including six adrenocortical carcinomas, three pheochromocytomas, three ganglioneuroma lesions, two lymphomas, two adenomas, and two tuberculomas. Table 2 presents the baseline characteristics of patients categorized by diagnosis.

**Table 2:** Baseline characteristics of patients stratified by diagnosis

Diagnosis	Number of patients	Male patients (number (%))	Female patients (number (%))	Mean age (years)	Age range (years)
Adrenal adenomas	70 (81.4%)	20 (28%)	50 (72%)	38	19-82
Adrenocortical carcinomas	6 (7%)	3 (50%)	3 (50%)	55	44-68
Pheochromocytomas	3 (3.5%)	1 (33%)	2 (67%)	42	29-55
Ganglioneuromas	3 (3.5%)	2 (67%)	1 (33%)	35	20-50
Lymphomas	2 (2.3%)	1 (50%)	1 (50%)	40	30-50
Tuberculomas	2 (2.3%)	1 (50%)	1 (50%)	36	28-42

**Figure 3:**
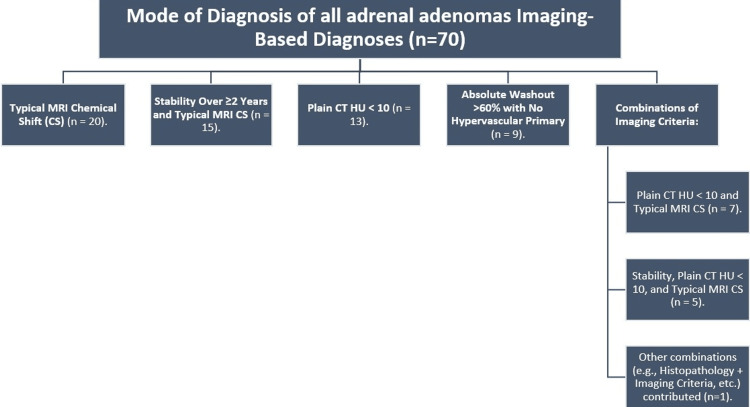
Flowchart illustrating the diagnostic methods for 70 adrenal adenomas, including standalone imaging criteria (e.g., MRI chemical shift, CT HU < 10) and combinations of imaging findings, clinical stability, and histopathology MRI: magnetic resonance imaging, CS: chemical shift, CT: computed tomography, HU: Hounsfield units

The remaining 70 adrenal lesions were confirmed as adenomas using various imaging criteria. These criteria included typical MRI chemical shift (n = 20), stability over ≥2 years combined with typical MRI chemical shift (n = 15), plain CT attenuation with HU values < 10 (n = 13), and absolute washout of >60% with no hypervascular primary tumors (n = 9), as well as combinations of imaging criteria, such as plain CT HU < 10 with typical MRI chemical shift (n = 7) and stability combined with plain CT HU < 10 and typical MRI chemical shift (n = 5). A single case involved a combination of histopathology and imaging findings (Figure 3).

The quantitative assessment of the adrenal lesions revealed that 10 out of 86 lesions were larger than 5 cm in diameter. Among these larger lesions, seven were adrenocortical carcinomas, two were pheochromocytomas, and one was a pathology-proven adenoma. The size of adrenal lesions showed a sensitivity of 98% and a specificity of 56% in distinguishing adenomas from other adrenal lesions.

In the qualitative assessment of signal intensity homogeneity, 46 out of 86 lesions exhibited homogenous signal intensity. Among these lesions, 45 were adenomas, and only one was a pheochromocytoma. The homogeneity of signal intensity demonstrated high specificity (94%) but relatively lower sensitivity (60%) in identifying adenomas.

The chemical shift imaging (CSI) analysis revealed that a signal drop of more than 16.5% was observed in 64 adenomas (out of 70 adenomas), one pheochromocytoma, and one adrenocortical carcinoma (ACC). No significant signal drop was observed in six adenomas, two pheochromocytoma lesions, six ACC, two lymphomas, two ganglioneuroma lesions, and two tuberculoma lesions. The sensitivity and specificity of CSI in distinguishing adenomas from other adrenal lesions were 91% and 87.5%, respectively.

Regarding the time to peak values, the sensitivity and specificity in differentiating adenomas from other adrenal lesions were 63% and 81%, respectively. A time to peak of less than 80 seconds was observed in 47 lesions, 44 of which were adenomas and three of which were pheochromocytomas.

The five-minute washout criterion, defined as a signal intensity drop of more than 25% compared to the one-minute phase, showed a specificity of 75% and a sensitivity of 60% in identifying adenomas.

Table 3 provides insights into the performance of various imaging features and scoring systems in differentiating adrenal adenomas from other adrenal lesions.

**Table 3:** Diagnostic performance of individual MRI features and the total mpMRI score in differentiating adrenal adenomas from other adrenal lesions, including sensitivity, specificity, 95% CIs, PPV, and NPV MRI: magnetic resonance imaging, mpMRI: multiparametric magnetic resonance imaging, CIs: confidence intervals, PPV: positive predictive value, NPV: negative predictive value, T2WI: T2-weighted imaging, CSI: chemical shift imaging

Image characteristic	Sensitivity (%)	95% CI	Specificity (%)	95% CI	PPV (%)	NPV (%)
Size < 5 cm	98	91.0-99.9	56	30.6-78.5	85	85
T2WI homogeneous signal	60	47.1-71.7	94	70.6-99.7	92	66
CSI positive	91	82.4-96.3	88	62.2-97.9	89	89
Time to peak < 80 seconds	63	50.2-74.5	81	54.4-96.0	77	70
5-minute washout > 25%	60	47.1-71.7	75	47.6-92.7	72	64
Total MRI score ≥ 3	98.6	92.5-100.0	93.8	69.8-99.8	98.6	93.8

Diagnostic performance of MRI features and scoring system

The mpMRI scoring system was evaluated in a cohort of 86 adrenal lesions, using a score threshold of 3 or higher to indicate an adrenal adenoma. The gold standard, defined as the confirmed diagnosis through histopathology, imaging findings, and clinical follow-up, identified 70 adrenal adenomas and 16 non-adenomas. The mpMRI scoring system correctly identified 69 true positives and 15 true negatives, achieving a sensitivity of 98.6% and a specificity of 93.8%. Among the 70 suggested adenomas, only one was missed by mpMRI due to atypical imaging characteristics, primarily in a lipid-poor adenoma. One lesion was misclassified as an adenoma due to overlapping imaging features, resulting in a single false positive. The calculated diagnostic parameters included a positive predictive value (PPV) of 98.6% and a negative predictive value (NPV) of 93.8%. Furthermore, the Chi-square test demonstrated a statistically significant association between the mpMRI scoring system and the gold standard, with a Chi-square value of 60.46 and a p-value of less than 0.0001, reinforcing the substantial diagnostic accuracy of the system (Table 4).

**Table 4:** Comparison of mpMRI scoring system results with the gold standard This table compares the outcomes of the mpMRI scoring system with the gold standard for diagnosing adrenal lesions. mpMRI: multiparametric magnetic resonance imaging

mpMRI score	Gold standard positive	Gold standard negative	Total
Positive	69 (80.23%)	1 (1.16%)	70 (81.4%)
Negative	1 (1.16%)	15 (17.44%)	16 (18.6%)
Total	70 (81.4%)	16 (18.6%)	86 (100.0%)

## Discussion

The current study presents a novel multiparametric MRI-based scoring system to distinguish adrenal adenomas from other adrenal lesions, with a retrospective analysis of 77 patients over a decade. The discussion elaborates on the findings, addresses limitations, and contextualizes the study within existing literature.

The strong diagnostic performance of the mpMRI scoring system highlights its value as a non-invasive tool for distinguishing adrenal lesions (Table 4). Among the parameters evaluated, chemical shift imaging (CSI) was given greater weight due to its superior sensitivity and specificity in identifying lipid-rich adenomas [4,5], emphasizing its pivotal role in the scoring framework. Additional features, including lesion size, T2-weighted signal homogeneity, time to peak enhancement, and dynamic contrast washout, have been individually described in previous literature (detailed in the Introduction section) as valuable diagnostic tools for differentiating adrenal lesions. However, these parameters have not been previously combined into a unified scoring system.

**Figure 4:**
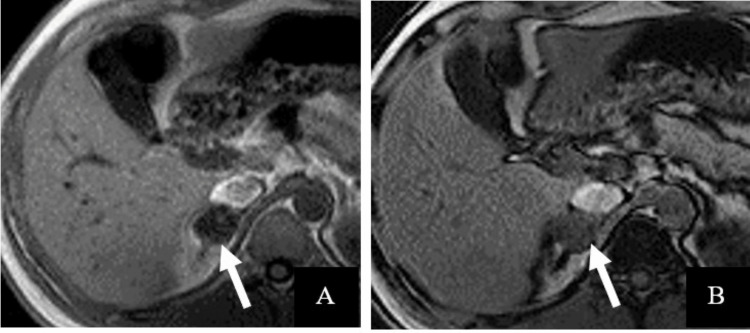
Axial 2D gradient-echo T1-weighted MRI for a 33-year-old man with histopathology-proven pheochromocytoma In-phase (A) and opposed-phase (B) images show diffuse homogeneous objective signal loss within the lesion with a mean value of 101 SI in the in-phase image and 35 in the opposed-phase image, with an SI index of 65% (white arrows). MRI: magnetic resonance imaging, SI: signal intensity

False positives, where non-adenoma lesions were incorrectly classified as adenomas, accounted for 1.2% of cases (one out of 86 lesions). This false positive was a histopathology-proven pheochromocytoma (Figure 4), which was misclassified due to overlapping imaging characteristics, including a rare presence of microscopic fat, typically seen in lipid-rich adenomas. False negatives, representing 1.2% of cases (one out of 86 lesions), were attributed to a lipid-poor adenoma that lacked the typical imaging characteristics of lipid-rich adenomas. This lesion exhibited insufficient washout values and atypical imaging patterns, leading to its misclassification as a non-adenoma. These findings underscore the diagnostic challenges of rare imaging features in specific adrenal lesions.

**Figure 5:**
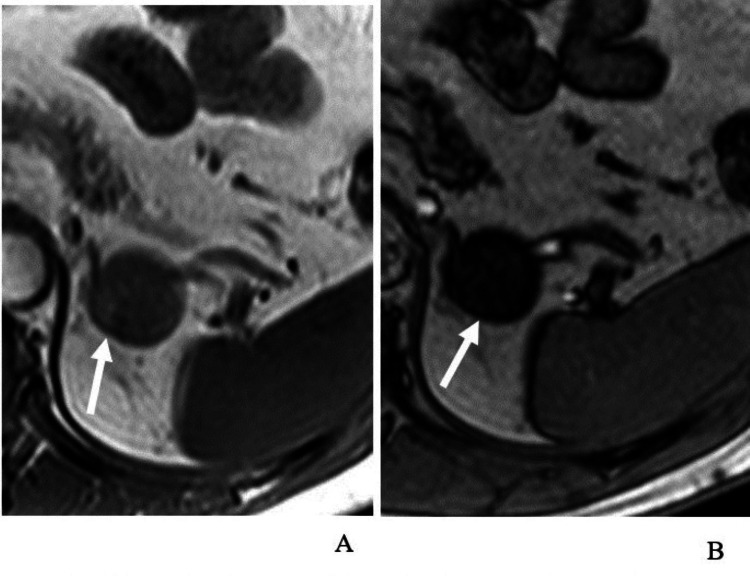
Axial 2D gradient-echo T1-weighted MRI for a 46-year-old man with an incidentally detected adrenal adenoma In-phase (A) and opposed-phase (B) images show diffuse homogeneous subjective signal loss within the adenoma, with a signal intensity index of 39% (white arrows). MRI: magnetic resonance imaging

The study also identified homogeneous T2 signal intensity as a novel and valuable parameter for differentiating adrenal adenomas from other lesions, particularly when combined with chemical shift imaging (CSI), lesion size, and dynamic contrast washout. Homogeneous T2 signal was strongly associated with benign adenomas in this cohort (Figure 5), with fewer overlap instances than lipid-poor malignancies, where heterogeneity was more frequently observed. Unlike previous studies, which primarily emphasized CSI [8] or CT attenuation values [11], this is the first to highlight T2 signal homogeneity as an independent diagnostic feature, addressing a critical gap in existing imaging approaches. This new observation enhances the scoring system's ability to classify adrenal lesions accurately.

It is worth mentioning that eliminating the time to peak and five-minute washout criteria from the multiparametric MRI scoring system does not significantly affect its ability to identify adrenal adenomas accurately. The system still correctly diagnosed 68 out of 70 confirmed adenomas, achieving a high accuracy of 97.3%, by focusing on key MRI features such as lesion size, chemical shift imaging, and homogeneity on T2-weighted imaging. Moreover, this scoring system can be safely applied to patients for whom contrast use is contraindicated, whether for MRI or CT scans, offering a reliable and non-invasive alternative for diagnosing adrenal adenomas (Table 5).

**Table 5:** Comparison of the mpMRI scoring system performance with and without time to peak and washout Parameters mpMRI: multiparametric magnetic resonance imaging, CI: confidence interval

Parameter	With time to peak and washout	Without time to peak and washout
Total number of adrenal lesions	86	86
Total number of confirmed adenomas	70	70
Adenomas correctly identified	69	68
False negatives (adenomas misclassified)	1	2
Sensitivity	98.6% (95% CI: 92.5-100.0%)	97.1% (95% CI: 89.8-99.6%)
Specificity	94.0% (95% CI: 69.8-99.8%)	94.0% (95% CI: 69.8-99.8%)
Accuracy	98.3% (95% CI: 91.1-99.9%)	97.3% (95% CI: 89.7-99.6%)

One of the primary limitations of this study is the reliance on imaging findings for diagnosis in most cases, with histopathological confirmation available for only 20.9% (18 out of 86 lesions) of cases, including just two confirmed adrenal adenomas. While this represents a potential limitation, it is a reflection of real-world clinical practice, where histopathology is not routinely pursued for adrenal lesions that are small and asymptomatic or exhibit benign imaging features [9]. The study flowchart (Figure 2) shows that adrenal adenomas were confirmed using a combination of diagnostic modalities, including CT and MRI findings. Combined with longitudinal follow-up in selected cases, these imaging modalities provided a reliable alternative to histopathology in differentiating benign adenomas from other lesions. Furthermore, the study's retrospective nature introduces inherent biases, including potential variability in imaging protocols, data completeness, and the absence of a standardized diagnostic pathway over the study period. Another limitation is the relatively small sample size, particularly for non-adenoma lesions, which may limit the generalizability of the findings. Finally, using traditional 15-minute delayed imaging could have improved the sensitivity and specificity of the scoring system. However, as this study is based on retrospective data, the imaging protocol was predetermined and could not be modified, which further highlights the challenges associated with retrospective analyses.

Despite its limitations, this study represents an essential contribution to the field. Previous studies have reported lower diagnostic sensitivity (approximately 71%) when applying radiological criteria retrospectively to histologically confirmed lesions, particularly for lipid-poor adenomas [10].

## Conclusions

This study presents a novel and practical multiparametric MRI (mpMRI) scoring system demonstrating high diagnostic accuracy in distinguishing adrenal adenomas from other adrenal lesions. Integrating key imaging parameters, including lesion size, chemical shift imaging, T2 signal homogeneity, time to peak enhancement, and delayed washout, into a single scoring framework, the system achieved a sensitivity of 98.6% and specificity of 93.8%. Including a homogeneous T2 signal as a strong predictor of benignity adds diagnostic value, particularly in cases where fat content alone is inconclusive.

Notably, the scoring system retained high accuracy even when contrast-enhanced sequences were excluded, suggesting its potential utility in patients with contrast contraindication. This flexibility enhances its applicability in real-world clinical settings, providing radiologists and referring clinicians with a structured and reproducible approach for characterizing adrenal lesions and reducing unnecessary interventions or follow-up imaging. However, since the scoring system was retrospectively derived and tested on the same dataset, incorporation bias may have influenced the reported accuracy. As a precursor to further research, this study highlights the need for multicenter prospective validation with histopathological correlation to refine and confirm the clinical utility of the scoring system.
